# Association of Plasma IL‐34, IL‐37, and IL‐38 With Asthma, Eczema, and Nasopharyngeal Bacteria in Early Childhood

**DOI:** 10.1111/apm.70281

**Published:** 2026-11-06

**Authors:** Johanna T. Teräsjärvi, Laura Toivonen, Jussi Mertsola, Ville Peltola, Qiushui He

**Affiliations:** ^1^ Institute of Biomedicine, Research Centre for Infections and Immunity University of Turku Turku Finland; ^2^ Department of Paediatrics and Adolescent Medicine Turku University Hospital and University of Turku Turku Finland; ^3^ InFLAMES Research Flagship Centre University of Turku Turku Finland

**Keywords:** bacteria, eczema, IL‐34, IL‐37, IL‐38 I asthma

## Abstract

Asthma and atopic dermatitis (AD) are common childhood inflammatory diseases linked to type 2 immune responses and early‐life environmental factors. Emerging evidence suggests that nasopharyngeal microbiota and immune‐regulatory cytokines may influence disease development. We investigated the association between plasma IL‐34, IL‐37, and IL‐38 levels at 24 months, nasopharyngeal bacterial colonization, and later asthma and eczema in a Finnish birth cohort (*n* = 145). Children with persistent eczema at 13 and 24 months had lower IL‐37 levels than children without eczema (*p* = 0.030). No associations were observed between cytokine levels and asthma at 7 years. Among children with nasopharyngeal culture data (*n* = 35), those colonized at 24 months with at least one of the following bacterial species: *Streptococcus pneumoniae* , *Moraxella catarrhalis* , *Staphylococcus aureus* , or *Haemophilus influenzae* , were associated with significantly lower IL‐34 (*p* = 0.015) and IL‐37 (*p* = 0.006) levels. These findings suggest that IL‐34 and IL‐37 may be involved in immune responses associated with nasopharyngeal bacterial colonization and eczema during early childhood.

## 1 Introduction

Asthma and eczema are chronic inflammatory diseases frequently emerging in early childhood [1]. Both are associated with allergic sensitization and type 2 immune responses [1]. In asthma, Th2‐derived cytokines such as IL‐4 and IL‐13 promote IgE production, and increase mucus secretion [1], while IL‐5 plays a central role in eosinophil maturation, activation, survival, and recruitment to the airways [2]. Pediatric eczema is characterized by early Th2 dominance, with additional involvement of Th17, Th22, and Th9 cells [1]. Skin barrier dysfunction, genetic susceptibility, microbiome changes, and environmental triggers all contribute to the progression from eczema to other allergic diseases [1].

Three recently identified immunoregulatory cytokines have emerged as potential contributors to allergic diseases, including asthma and eczema [3, 4, 5]. Among these, IL‐37 and IL‐38 belong to the IL‐1 cytokine superfamily, which comprises 11 cytokines that plays a pivotal role in coordinating immune and inflammatory responses [6]. All these cytokines share structural and functional similarities with Toll‐like receptors (TLRs), and the 11 members of the IL‐1 family are classified into three subfamilies: the IL‐1, IL‐18, and IL‐36 subfamilies [6].

IL‐37, belonging to the IL‐18 subfamily, and IL‐38, belonging to the IL‐36 subfamily, both exhibit anti‐inflammatory properties [6]. IL‐37 suppresses inflammatory responses by inhibiting pro‐inflammatory cytokine production [7]. In the pathogenesis of asthma, IL‐37 can promote IL‐10 expression and inhibit the increase of Th2 cytokines such as IL‐4, IL‐6, and IL‐13, and the level of inflammatory IL‐17 in lung homeostasis [8]. Decreased IL‐37 expression has been reported in the skin barrier of children with atopic dermatitis (AD) [3]. In addition, Hou et al. demonstrated that IL‐37b can reduce eosinophil‐driven allergic inflammation by regulating autophagy, gut bacterial diversity, and related metabolites in AD [9]. IL‐38 functions as an accessory IL‐36 receptor antagonist [6], reducing Th17‐associated cytokine secretion and alleviating airway remodeling in chronic asthma [4].

IL‐38 shares high structural similarity with IL‐36 and functions as an accessory IL‐36 receptor antagonist [10]. By binding to the IL‐36R, it can decrease the secretion of asthma‐related Th17 cytokines, IL‐22, and alleviate airway remodeling in chronic asthma [4]. Notably, IL‐38 levels have been reported to correlate negatively with IL‐10 levels in children with asthma [11, 12]. Furthermore, studies in murine models suggest that IL‐38 may contribute to airway eosinophilic inflammation in asthma by inducing IL‐5 [11, 12].

In contrast, IL 34 is not a member of the IL‐1 family but is important for skin immune homeostasis [5]. IL‐34 regulates the survival, proliferation, and differentiation of monocytes, macrophages, and dendritic cells through its interaction with the colony‐stimulating factor‐1 receptor (CSF‐1R) [13]. It is predominantly expressed in the epidermis, with lower levels in the dermis [13] where it is essential for the maintenance of Langerhans cells, the major antigen‐presenting cells of the skin [5]. Reduced IL‐34 expression has been reported in several inflammatory skin disorders, including AD, eczema, and psoriasis, suggesting a role in maintaining skin immune homeostasis and barrier‐associated immune regulation [5].

In parallel with cytokine‐mediated mechanisms, early‐life nasopharyngeal (NP) microbiota composition has been shown to shape immune maturation and influence susceptibility to allergic diseases [14]. Colonization with *Haemophilus influenzae* and *Moraxella catarrhalis* has been linked to asthma [15], while *Staphylococcus aureus* is more commonly associated with eczema [14].

Here, we focused on three recently described immunoregulatory cytokines, IL‐34, IL‐37, and IL‐38, which have been reported in asthma, atopic dermatitis, and other inflammatory skin diseases, suggesting a potential role in the development and regulation of allergic inflammation [3, 4, 5]. We studied whether early‐life NP colonization is associated with altered levels of these cytokines, and whether their concentrations are linked to subsequent development of eczema or asthma.

## 2 Materials and Methods

The study was conducted within the Finnish STEPS birth cohort in children born between January 2008 and April 2010 in the Hospital District of Southwest Finland [16]. Altogether, 923 children were intensively monitored for respiratory tract infections from birth to 2 years of age and were subsequently followed through annual questionnaires and health registry data [15]. Physician‐diagnosed asthma at 7 years was defined as either a documented diagnosis in medical records or an electronic prescription for inhaled corticosteroids for asthma [15]. Eczema was defined based on ISAAC questionnaires, and children classified as having eczema in this study had a reported eczema diagnosis at both 13 and 24 months of age. Nasopharyngeal and blood samples were collected during scheduled visits at ages 2, 13, and 24 months [15]. All children with physician‐diagnosed asthma (*n* = 37) were included, along with 108 randomly selected controls without asthma. Plasma IL‐34, IL‐37, and IL‐38 levels were measured in samples collected at 2 years of age using the MILLIPLEX Human Cytokine Panel 4 (Merck Millipore, Burlington, Massachusetts, US) on the Luminex platform. The detection limits were 69.0 pg/mL, 2 pg/mL, and 9.5 pg/mL, respectively. Cytokine concentrations below the detection limit were assigned to half of the detection limit for statistical analyses.

Nasopharyngeal samples were cultured on four selective media plates, and bacterial species were identified using standard phenotypic methods [17]. All bacteria were identified at least to the genus levels. However, only *S. pneumoniae* , *S. aureus* , *H. influenzae*, and *M. catarrhalis* were included in the species‐specific analyses because these bacteria have been consistently linked to allergic diseases and asthma in previous studies [12].

Statistical analyses were performed using SPSS (version 30.0, IBM Corp. NY, US). The non‐normally distributed data were compared by Mann–Whitney *U* test, and correlation between cytokine levels and presence of asthma was analyzed by binary logistic regression analysis. Sex, delivery mode, existence of older siblings, duration of breast‐feeding, child's eczema, and parental asthma were included as confounders.

## 3 Results

In this subset of 145 children, 65.5% were male and 34.5% were female. Vaginal delivery was reported for 86.8% of children, and 65.7% were exclusively or partially breastfed until 6 months of age. At the time of sampling (24 months), information on diagnosis of eczema was available in 129 children. Of them, 34 (26.6%) had a diagnosis of eczema, and of these 10 were newly diagnosed and 24 had eczema also at 13 months of age. Physician‐diagnosed asthma was documented in 37 children (25.5%) by 7 years of age.

As shown in Figure 1, IL‐34, IL‐37, and IL‐38 concentrations at 24 months were compared by eczema status. Children with eczema at 13 and 24 months of age had lower plasma IL‐37 levels at 2 years of age (6.69 pg/mL; Q1:1.00, Q3:46.55) compared with children without eczema (23.64; Q1: 4.07, Q3: 97.15) (*p* = 0.032, adjusted *p*(pa) = 0.192). However, IL‐34 and IL‐38 were not associated with eczema. None of the measured cytokines at age of 2 years differed between children who later developed asthma and those who did not (Figure 1).

**FIGURE 1:**
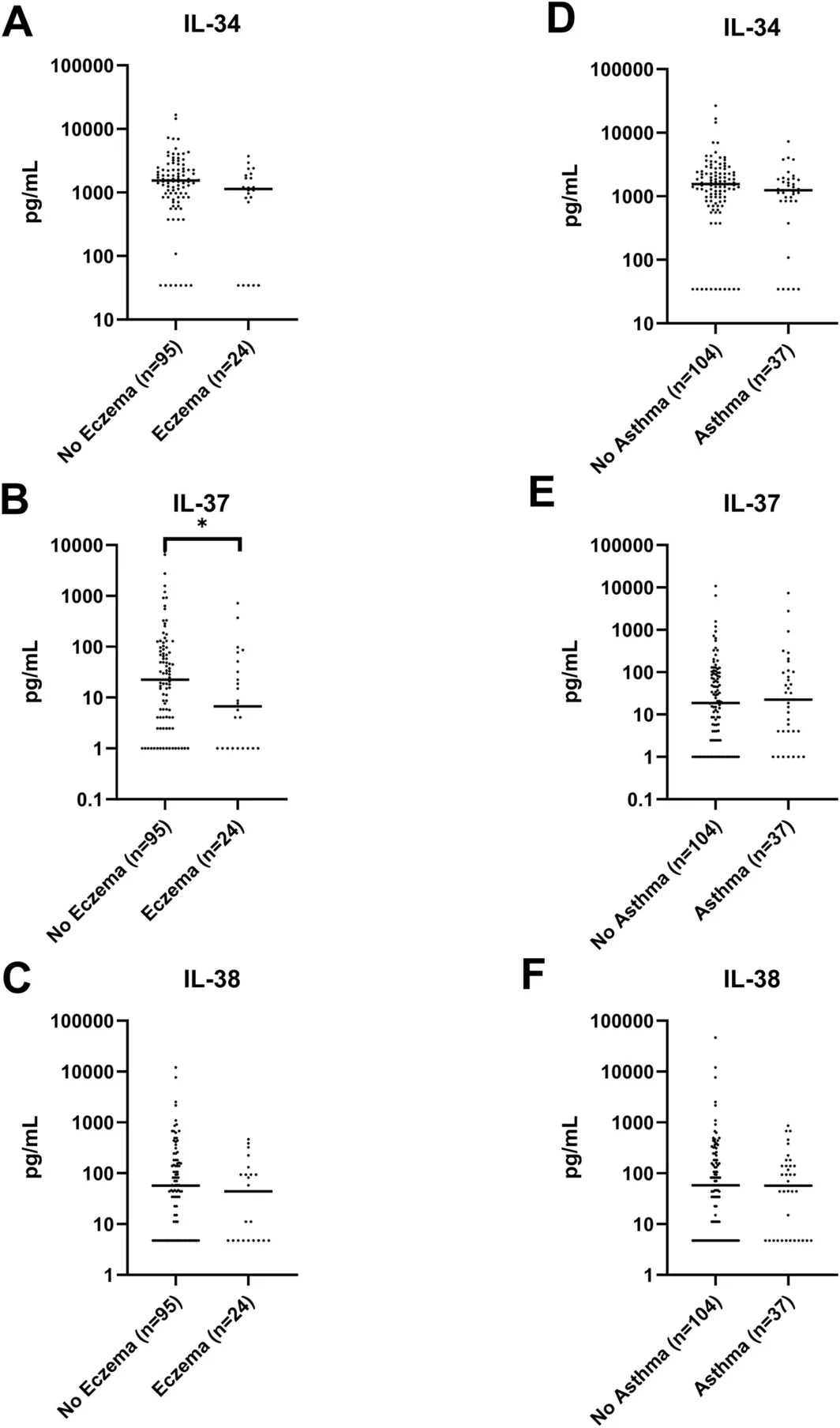
Plasma concentrations of IL‐34, IL‐37, and IL‐38 (pg/mL) in children with and without eczema (A–C) and with and without asthma (D–F). Each dot represents an individual participant, and horizontal lines indicate group medians. Values below the detection limit were assigned half of the detection limit for visualization and statistical analyses. (A–C) No differences in IL‐34 (A) or IL‐38 (C) levels were observed between children with and without eczema. IL‐37 levels were elevated in children without eczema compared to those with eczema (*p = 0.039, Pa = 0.192). (D–F) No significant differences in IL‐34 (D), IL‐37 (E), or IL‐38 (F) concentrations were detected between individuals with and without asthma.

In the subset of children with nasopharyngeal culture data (*n* = 35), those colonized by at least one of the following bacteria, *S. pneumoniae* , *M. catarrhalis* , *S. aureus* , or *H. influenzae* , at 24 months (*n* = 28) had significantly lower levels of IL‐34 (*p* = 0.015) and IL‐37 (*p* = 0.006) than noncolonized children (*n* = 7) (Figure 2), with IL‐38 showing similar but nonsignificant results.

**FIGURE 2:**
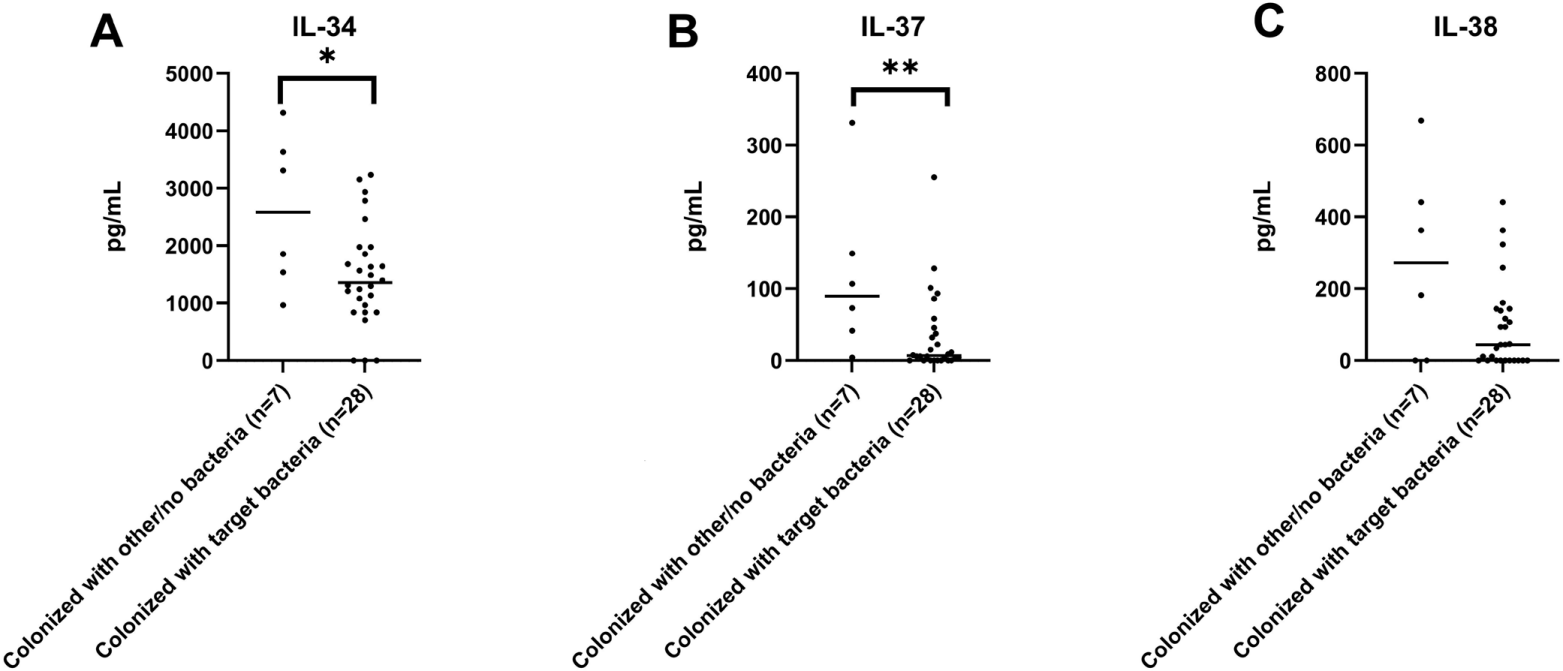
Concentrations of IL‐34 (A), IL‐37 (B), and IL‐38 (C) in plasma samples from children colonized with at least one of four target bacterial species and children colonized with other bacteria or no bacteria. The four target bacterial species were: *Streptococcus pneumoniae* , *Staphylococcus aureus* , *Haemophilus influenzae* , or *Moraxella catarrhalis* . The other nasopharyngeal bacteria were identified at the genus levels. Horizontal lines indicate medians. Statistical significance was assessed using the Mann–Whitney *U* test (*p < 0.015, **p < 0.006).

## 4 Discussion

This study demonstrates an association between NP carriage of at least one of the pathogenic bacteria ( *S. pneumoniae* , *M. catarrhalis* , *S. aureus* or *H. influenzae* ) and plasma IL‐37 and IL‐34 concentrations. In addition, we found lower plasma IL‐37 levels in children with eczema, in line with findings by Zhou et al., who reported downregulated *IL1F7* (IL‐37) gene expression and decreased epidermal IL‐37 protein levels in AD lesions. Notably, they found that IL‐37 expression was positively associated with the skin barrier proteins filaggrin (FLG) and FLG‐2, supporting its role in homeostasis [18]. In contrast to their potential involvement in eczema and bacterial colonization, none of the measured cytokines was associated with physician‐diagnosed asthma at 7 years. Prior studies have reported downregulated IL‐37 levels in asthma and a possible pro‐inflammatory role of IL‐38 in airway eosinophilia [6, 19, 20].

Our previous work in this same cohort showed that lower sST2 levels in early childhood were associated with the later development of asthma [21]. As a decoy receptor for IL‐33, sST2 modulates IL‐33 signaling, and reduced sST2 concentrations may reflect enhanced IL‐33 bioavailability and downstream activity [6]. IL‐33 is a type‐2–promoting cytokine, whereas IL‐37 and IL‐38 differ functionally by participating predominantly in Th1‐related and regulatory pathways [6]. Although all three belong to the IL‐1 family, IL‐33 is more directly implicated in asthma pathogenesis, while IL‐37 and IL‐38 are considered more relevant to antimicrobial defense and the regulation of inflammatory responses [6].

Interestingly, children colonized with at least one of the four target nasopharyngeal bacterial species had lower circulating IL‐37 and IL‐34 concentrations than non‐colonized children. The biological mechanisms underlying this finding remain to be studied, as both cytokines are involved in the regulation of immune homeostasis and anti‐inflammatory responses, suggesting that reduced levels may reflect altered host–microbe interactions at mucosal surfaces [3, 5, 8, 9]. IL‐37 is a potent anti‐inflammatory cytokine that suppresses innate and adaptive immune responses and promotes regulatory immune pathways, including regulatory T‐cell activity [22]. Experimental studies have also suggested a role for IL‐37 in host‐microbiota interactions, as IL‐37 expression has been associated with modulation of microbial diversity and restoration of immune homeostasis [9]. Thus, the lower IL‐37 concentrations observed in bacteria colonized children may reflect differences in host‐microbial interactions and immune maturation associated with bacterial colonization rather than active inflammatory responses.

IL‐34 is highly expressed in epithelial tissues, particularly the skin, and has been implicated in local immune regulation as well as the maintenance of tissue‐resident monocytes and macrophages [5]. However, despite the biological relevance of IL‐34 in skin immunity, we found no association between circulating IL‐34 concentrations and eczema. Instead, lower IL‐34 levels were associated with nasopharyngeal bacterial colonization, suggesting that circulating IL‐34 may be more reflective of host–microbe interactions and immune homeostasis than of clinical allergic manifestations. As bacterial colonization differs from active infection, the observed lower IL‐34 concentrations may further indicate the absence of substantial systemic inflammatory activation.

The study has limitations, including small sample size, particularly for NP colonization analyses. Plasma cytokine concentrations may not fully reflect mucosal immune responses in the skin or airways.

In conclusion, early life nasopharyngeal colonization with common commensal bacteria was associated with lower levels of plasma IL‐34 and IL‐37, suggesting an interaction between bacterial carriage and systemic regulatory cytokine responses. Although lower IL‐37 was observed in children with eczema, neither cytokine levels nor NP colonization independently predicted eczema or asthma in adjusted analyses.

## Funding

This work was supported by the Tampere Tuberculosis Foundation (26006205 to Q.H.), Sigrid Juselius Foundation (260050 to Q.H.), the University of Turku Foundation, the Research Foundation of the Pulmonary Diseases, State research funding for the Western Finland cooperation area, the Academy of Finland, and the Foundation for Pediatric Research. The funders had no role in the study design, data collection, analysis, interpretation of the results, manuscript preparation, or the decision to publish.

## Ethics Statement

The Ministry of Social Affairs and Health and the Ethics Committee of the Hospital District of Southwest Finland have approved the STEPS Study (27 February 2007). Written informed consent was obtained from the parents. They were informed of their right to withdraw from the study at any point.

## Consent

The participants were informed that the data may be used in scientific publications, and that all reported information would be handled confidentially and presented in a way that prevents identification of individual participants.

## Conflicts of Interest

The authors declare no conflicts of interest.

## Data Availability

The data that support the findings of this study are available from the corresponding author upon reasonable request.
